# Clinical Benefits of *n*-3 PUFA and ɤ-Linolenic Acid in Patients with Rheumatoid Arthritis

**DOI:** 10.3390/nu9040325

**Published:** 2017-03-25

**Authors:** Mirjana Veselinovic, Dragan Vasiljevic, Vesna Vucic, Aleksandra Arsic, Snjezana Petrovic, Aleksandra Tomic-Lucic, Maja Savic, Sandra Zivanovic, Vladislava Stojic, Vladimir Jakovljevic

**Affiliations:** 1 Department of Internal medicine, Faculty of Medical Sciences, University of Kragujevac, Kragujevac 34000, Serbia; veselinovic.m@sbb.rs (M.V.); sanlusa@ptt.rs (A.T.-L.); 2 Department of Hygiene, Institute for Public Health, Faculty of Medical Sciences, University of Kragujevac, Kragujevac 34000, Serbia; dvg_gana@yahoo.com; 3 Centre of Research Excellence in Nutrition and Metabolism, Institute for Medical Research, University of Belgrade, Beograd 11000, Serbia; vesna.vucic.imr@gmail.com (V.V.); vesna.vucic.imr@gmail.com (A.A.); snjezana570.imr12@gmail.com (S.P.); 4 Department of Pharmacy, Faculty of Medical Sciences, University of Kragujevac, Kragujevac 34000, Serbia; maja.jovanovic.2008.38@gmail.com; 5 Faculty of Hotel Management and Tourism, Department of Natural Sciences and medicine, University of Kragujevac, Kragujevac 34000, Serbia; zivanovicsandra@hotmail.com; 6 Faculty of Medical Sciences, University of Kragujevac, Kragujevac 34000, Serbia; vladisavastojic@medf.kg.ac.rs; 7 Faculty of Medical Sciences, University of Kragujevac, Physiology, Kragujevac 34000, Serbia

**Keywords:** *n*-3 PUFA, ɤ-linolenic acid, rheumatoid arthritis

## Abstract

(1) Background: Marine *n*-3 polyunsaturated fatty acids (PUFA) and ɤ-linolenic acid (GLA) are well-known anti-inflammatory agents that may help in the treatment of inflammatory disorders. Their effects were examined in patients with rheumatoid arthritis; (2) Methods: Sixty patients with active rheumatoid arthritis were involved in a prospective, randomized trial of a 12 week supplementation with fish oil (group I), fish oil with primrose evening oil (group II), or with no supplementation (group III). Clinical and laboratory evaluations were done at the beginning and at the end of the study; (3) Results: The Disease Activity Score 28 (DAS 28 score), number of tender joints and visual analogue scale (VAS) score decreased notably after supplementation in groups I and II (*p* < 0.001). In plasma phospholipids the *n*-6/*n*-3 fatty acids ratio declined from 15.47 ± 5.51 to 10.62 ± 5.07 (*p* = 0.005), and from 18.15 ± 5.04 to 13.50 ± 4.81 (*p* = 0.005) in groups I and II respectively. The combination of *n*-3 PUFA and GLA (group II) increased ɤ-linolenic acid (0.00 ± 0.00 to 0.13 ± 0.11, *p* < 0.001), which was undetectable in all groups before the treatments; (4) Conclusion: Daily supplementation with *n*-3 fatty acids alone or in combination with GLA exerted significant clinical benefits and certain changes in disease activity.

## 1. Introduction

Rheumatoid arthritis (RA) is one of the most common autoimmune disorders. The main characteristic of RA is the onset of pathological changes in the lining of the joints. Due to the infiltration of macrophages, B cells, and CD4+ helper T cells into the synovial stroma, synovium proliferates causing swelling and pain of joints [1,2]. Furthermore, the overproduction of inflammatory molecules, prostaglandin E2 (PGE2), tumour necrosis factor (TNF), interleukin (IL)-1, and cytokines induces chronic inflammation [3,4]. Especially IL-1 and TNF play important roles in inflammation during RA. Numerous randomized controlled clinical trials have reported a strong anti-inflammatory potential of marine *n*-3 long-chain polyunsaturated fatty acids (*n*-3 LC-PUFAs) and improved clinical parameters of RA [5,6]. These effects are mostly attributed to eicosapentaenoic acid (EPA, C20:5 *n*-3) and docosahexaenoic acid (DHA, C22:6 *n*-3) acting in two ways. Firstly, they interfere with the enzymatic conversion of arachidonic acid (AA, C20:4 *n*-6) to pro–inflammatory prostaglandins (PGs) and leukotrienes (LTs). Secondly, EPA is a direct precursor in biosynthetic pathway of anti-inflammatory PGs (series-3) and LTs (series-5). Dietary *n*-3 LC-PUFAs replace AA in the phospholipid bilayer of the cell and then alter the membrane composition and fluidity, as well as cell signalling, gene transcription and metabolism of proresolving mediators [7,8,9,10]. Specialized proresolving mediators (resolvins, protectins and maresins) are bioactive lipid regulators implicated in the termination of inflammation by acting through specific G protein-coupled receptors. The resolution of inflammation is mediated by the termination of neutrophil recruitment, stimulation of apoptotic cells phagocytosis by macrophages, and their subsequent leaving of inflammatory tissue [11,12,13,14]. These novel lipid mediators provide rationale for further research on the beneficial effects of fish-oil enriched diets. 

Unlikely other *n*-6 PUFAs, ɤ-linolenic acid (C18:3*n*-6, GLA), found mostly in borage, black current seed and evening primrose oil (EPO), exerts anti-inflammatory activity. Namely, it can be converted to dihomo-ɤ-linolenic acid (C20:3*n*-6; DGLA) and further to PGs series-1, another group of eicosanoids with potential anti-inflammatory and immunoregulatory effects. Although these oils also contain linoleic acid (C18:2 *n*-6, LA), a precursor of AA, and some dietary GLA can eventually form AA, studies have shown that anti-inflammatory effects of EPO intake is prevailing [6]. Thus, GLA may also alleviate inflammation and disease severity in patients with RA, as shown in clinical trials using high doses of 1400–2800 mg GLA per day [15,16,17,18,19,20,21,22,23].

Although many studies have addressed fish oil supplementation in RA patients, the results are inconsistent. Large doses of at least 2.7 g EPA + DHA are likely required to achieve desirable outcomes in RA, but these doses can increase the risk of bleeding [24]. In this study, RA patients were supplemented either with *n*-3 PUFA or *n*-3 PUFA and GLA to examine possible anti-inflammatory effects and clinical benefits compared to the control group of patients, who had no supplementation. We also tested the possible synergistic effect of the combined *n*-3 PUFA + GLA supplementation. Our primary outcome measures were the alterations of PUFA in plasma phospholipids, with emphasis on EPA, DGLA and AA, that would indicate changes in the eicosanoids generation. The secondary outcome measure was the level of disease activity as measured with the DAS 28 disease activity score and the visual analogue scale (VAS).

## 2. Materials and Methods 

This prospective, double-blind randomized-controlled trial included 60 female patients (mean age 63.1 ± 9.6 years) with RA, recruited at the Rheumatology Department, Clinical Center Kragujevac, Serbia, in 2014. RA was diagnosed based on the American College of Rheumatology 2010 revised criteria [25]. The mean duration (± standard deviation) of the disease was 59 ± 60 months (12–180 months). All patients had the same anti-rheumatic therapy. Changes in the treatment regime (dose/type of drug, etc.) were predetermined exclusion criteria. Patients received low doses of corticosteroids (<10 mg/day), oral methotrexate (mean dose 15 mg/week) and folic acid 10 mg/week, while nonsteroidal anti-inflammatory drugs (NSAIDs) were given occasionally. Patients were eligible if the dosage of NSAIDs and/or corticosteroids had been constant for at least one month at the recruitment, and remained unchanged during the study, while the dose of disease-modifying antirheumatic drugs (DMARDs) had to be stable for at least two months before and throughout the trial. 

Patients were randomly allocated to three groups of 20 patients. The first group was taking 5 g of fish oil (5 Omega-3 Cardio^®^ gel capsules, Natural Wealth, NBTY Inc., New York, NY, USA; each containing 1 g of concentrated fish oil with 300 mg of DHA, 200 mg of EPA, and 100 mg of other *n*-3 PUFAs) daily after meals. The second group received 2 Omega-3 Cardio^®^ gel capsules and two Evening Primrose Oil^®^ gel capsules (Natural Wealth, NBTY Inc., New York, NY, USA; one gel capsule contains 1300 mg of evening primrose oil (Oenotherabiennis, seed) with 949 mg of LA and 117 mg of GLA) daily after meals, and the third group received only the previously described rheumatologic therapy, representing the control group. 

For the clinical evaluation of RA, we used the disease activity score (DAS) 28, which includes the 28 different joints for calculation (proximal interphalangeal joints, metacarpophalangeal joints, wrists, elbows, shoulders, and knees). The DAS 28 also takes into account the erythrocyte sedimentation rate (ESR) and the visual analogue scale (VAS) score. The VAS used a 100-mm horizontal scale where patients were asked to report the current pain intensity, by placing a line on a VAS scale between “no pain” (left end, 0 mm) and “excruciating pain” (right end 100 mm). The DAS 28 score calculation was performed by the automatic DAS 28 calculator V1.1-beta [26]. 

Laboratory tests and clinical evaluations were performed on two occasions: before the treatments were administered and three months after the beginning of the study. No patients dropped out from the study and compliance was checked at the end of each month by the researchers, who counted the number of capsules.

### 2.1. Adverse Effects

Adverse effects related to the study treatment were mild and their rate was similar in the two intervention groups. Mild gastrointestinal discomfort (mild diarrhoea, abdominal pain, dyspepsia or nausea lasting less than 72 h) that did not require any additional intervention, was noted in two patients in the first group.

The following exclusion criteria were used: serious chronic renal, liver or heart diseases, diabetes mellitus, smoking habit (in the last five years), uncontrolled hypertension, hyperlipidaemia, a family history of cardiovascular disease, premature menopause, a severe folic acid, vitamin B6 or vitamin B12 deficiency, and other forms of arthritis apart from RA. Clinical evaluations included anthropometric measurements (height, weight). Standing height was measured without shoes on a wall-mounted stadiometer (Perspective Enterprises, Kalamazoo, MI, USA). Body weight, body mass index (BMI), and body fat percent were measured with a Tanita body composition analyser (TBF-300, Tanita Corp., Tokyo, Japan). Written informed consent was obtained from all study participants prior to the enrolment. 

The study was approved by the Ethical Committee, Clinical Center, Kragujevac, Serbia, No 01/543-21.01.2014. This study has been conducted in accordance with the principles of the Declaration of Helsinki.

### 2.2. Fatty Acid (FA) Extraction and Analysis 

Venous blood samples were taken after 12 h of fasting at the beginning of the study and after 12 weeks of intervention. The method using a chloroform to methanol mixture (2:1 *v*/*v*) as a solvent and 2,6-di-tert-butyl-4-methylphenol (BHT, 10 mg/100 mL) as an antioxidant, was applied to extract total plasma lipids, as described previously [27]. In the next step, the phospholipid (PL) fraction was separated from other lipid subclasses via silica thin-layer chromatography in a neutral solvent system (petrol ether: diethyl ether: acetic acid, 87:12:1 *v*/*v*). The phospholipids fraction was placed in a screw-capped glass test tubes for methylation. The FA methyl esters were prepared as described previously [28] with slight modifications. In the test tube was added 1.5 mL hexane and 0.2 mL of 2 N NaOH in methanol and heated at 85°C for 1 h, and then 0.2 mL of 1M H_2_SO_4_ in methanol and heated at 85°C for 2 h. After cooling to room temperature and centrifugation on 1860× *g*, for 15 min, the hexane layer was dried under a stream of nitrogen. Prepared methyl esters were dissolved in 10 μL of hexane. Fatty acid (FA) analysis was started with the injection of 1 μL of sample into the gas-liquid chromatography device (Shimadzu GC 2014, flame ionization detector, Rtx 2330 column 60 m × 0.25 mm ID, film thickness 0.2 μm, Restek, Bellefonte, PA, USA). FA were identified according to the retention times of samples in comparison with the standards (Sigma Chemical Co., St. Louis, Missouri, MO, USA) and (PUFA)-2 standard mixture (Restek Co., Bellefonte, Pennsylvania, PA, USA), and expressed as relative percentages of total FA.

### 2.3. Statistical Analysis

Statistical data analysis was performed using SPSS 20.0 (SPSS Inc., Chicago, IL, USA), considering *p* ≤ 0.05 as significant. The results are presented as mean ± SD. The Kolmogorov-Smirnov test was applied to confirm the distribution of the data. Differences among the three tested groups at baseline and at the end of the study were assessed by one-way ANOVA followed by the Tukey post hoc test or by Kruskal-Wallis and Mann-Whitney U test, depending on the normality. To assess the effects of the supplementations, two-way (group and time) repeated measures ANOVA, was applied, with Bonferroni post hoc tests, where appropriate.

## 3. Results

Patients were randomly divided into three groups, which were receiving the same anti-rheumatic therapy. During 12 weeks, the first group took fish oil, the second one took fish oil + EPO and the third one did not take supplements. Patients’ basic characteristics are presented in Table 1. Age, BMI and the duration of RA were similar among the groups (Table 1). 

A significant decrease in the DAS 28 score was found in the fish oil group (4.99 ± 0.88 to 3.91 ± 0.80, *p* < 0.001, group I) and in the group II taking *n*-3 PUFA and EPO (4.76 ± 0.85 to 3.79 ± 0.72, group II). The DAS 28 score in group III trended towards a significant decrease (4.66 ± 0.80 to 4.23 ± 0.66, *p* = 0.053) (Table 2).

The number of painful joints and VAS score significantly decreased in both the supplemented groups (*p* ≤ 0.001) after 12 weeks, but not in the control group (Table 2).

Because of the high fish oil intake, EPA, DHA, *n*-3 PUFA and total PUFA increased significantly (*p* ≤ 0.05, Table 3) in plasma of the group I. Additionally, the *n*-6/*n*-3 PUFA ratio and MUFA, especially vaccenic acid (C18:1*n*-7) markedly decreased (Table 3).

In the group that ingested 2 g of fish oil + 2.6 g EPO/day (group II), the concentrations of GLA and AA (18:3 and 20:4 *n*-6) significantly increased, as well as *n*-3 EPA, DPA, DHA, *n*-3 PUFA, and total PUFA (Table 4). In line with this, level of SFA and the *n*-6/*n*-3 PUFA ratio were significantly lower after the supplementation (Table 4).

In the group III (patients without supplementation), no significant changes in FA proportions were found between baseline and the end of the trial (Table 5).

After 12 weeks of supplementation, when all groups were compared, levels of EPA, DHA and *n*-3 PUFA were higher, and the *n*-6 to *n*-3 ratio was lower in both supplemented groups than in the control patients. GLA and AA were higher in the group II (fish oil + EPO) than in the groups I and III (Table 6).

A repeated measures ANOVA revealed significant time × group interactions for the following FAs: stearic acid (18:0, *p* = 0.034), vaccenic acid (18:1*n*-7, *p* = 0.010), and MUFA (*p* = 0.028).

## 4. Discussion

Fatty acids found in plasma phospholipids reflect food intake over the last several days and weeks [29] as well as FA metabolism [30]. Altered FA composition was reported in many pathologic and physiological states [30,31,32,33], including chronic inflammation [34]. However, FA profiles and the course of diseases can be affected by PUFA supplementation [35,36]. In this study, the levels of supplemented PUFA in plasma increased in the groups I and II depending on the dosages (Table 3 and Table 4), demonstrating a good patient compliance and bioavailability of the supplements. The changes in FA composition in response to the supplementation (groups I and II) confirmed that plasma phospholipids are reliable biomarkers for fat intake (Table 3 and Table 4). In particular, it can be seen in Table 6, where the differences among the three groups after supplementation were most significant for FA used for supplementation.

The results have shown improvements in several laboratory and clinical parameters in RA patients with the active disease, who were taking anti-inflammatory oils along with the standard therapy. The consumption of 5 g of fish oil by the patients in group I resulted in significantly elevated EPA (Table 3). The AA content in phospholipids is a regulator of the biosynthesis of the proinflammatory prostanoids and leukotriens, whereas EPA has the opposite effect. Therefore, the AA/EPA ratio determines the degree of inflammation. Additionally, EPA and DHA support the synthesis of proresolving mediators such as protectins, resolvins, and maresins. Accordingly, a significant improvement in clinical variables, the severity of pain, the number of swollen/tender joints, as well as disease activity parameters (DAS 28 and VAS scores), were also observed in the supplemented group. 

In a 24-week study, Berbert et al. supplemented RA patients with 3 g/day of *n*-3 FA (1.8 g EPA and 1.2 g DHA) and found reduced pain and morning stiffness as well as better patient global assessment [37]. The others reported that daily supplementation of approximately 3 g *n*-3 PUFA for about three months was sufficient to achieve clinical improvements in these patients [38,39]. However, some meta-analyses have revealed no effect of *n*-3 PUFAs intake on the inflammation of joints, other clinical variables or the global assessment of RA patients [40,41]. In this regard, our study gives substantial contribution to recommendation for the use of *n*-3 PUFA in RA patients.

The combination of fish oil and EPO led to an increase in AA (Table 4). Namely, dietary GLA can be elongated to DGLA and subsequently desaturated to AA by delta-5 desaturase [42]. Hence, a slight increase of the AA content in phospholipids after GLA supplementation (group II; Table 4) was expected. In addition, LA as the main precursor for AA synthesis, was also presented in EPO capsules. Nevertheless, in our study supplementation with both fish oil and EPO resulted in significantly improved clinical variables, the severity of pain and the number of swollen and/or tender joints. These results indicate that the anti-inflammatory potential of GLA and DGLA, including the synthesis of PG series 1, are more pronounced than the conversion into inflammatory AA. 

The intake of *n*-6 LA was usually not controlled in studies on patients with RA. LA is ultimately converted into AA and further to pro-inflammatory cytokines [40]. PUFA of *n*-3 series can diminish this conversion by competitive inhibition [43]. It has also been proposed that restricting AA intake is required to benefit from the *n*-3 intake in RA patients [44]. Additional studies addressing plasma lipids and patient compliance measurements should confirm these findings.

Numerous investigations have shown the relationship between *n*-3 PUFA consumption and reducing inflammatory parameters [40,41,45,46]. Here we found lower ESR in group I and II than in the control patients, which was in line with other studies [37,47]. However, the link between *n*-3 PUFA and CRP is unclear. For instance, a 12-week supplementation with different doses of *n*-3 FA (1.5–6 g/day) did not decrease CRP concentrations when compared to the placebo group, as in our study [48]. It seems that inconsistent results come from different doses of *n*-3 PUFA needed for different inflammatory markers. Besides, we have shown that the consumption of fish oil combined with EPO also had positive effects in patients with RA, which were similar or even better (Table 2) than the fish oil supplementation alone. Thus, this combination has a great potential as an adjuvant therapy in patients with RA and other chronic inflammatory diseases.

Furthermore, chronic use of standard DMARDs, especially methotrexate, often leads to cardiovascular problems, that could be alleviated by concomitant consumption of *n*-3 PUFA [49]. As well, intake of 3 g of *n*-3 PUFA markedly increases the AA/EPA ratio and EPA + DHA index, which are highly reliable predictors of sudden cardiac death [50,51,52]. This is an added value, since patients with RA have strongly elevated risk of cardiac death [53,54]. Nevertheless, the exact impact of the control of chronic inflammation on the reduction of cardiovascular mortality should be elucidated [41].

The weakness of this study is the lack of a blinded placebo-controlled group, which would have made the interpretation of the data stronger. However, placebo capsules typically contain soy oil, corn oil or sunflower oil, which are all rich sources of *n*-6 PUFA. This could influence our results on FA profiles in the control group and must be taken into account in the interpretation of the FA alterations.

## 5. Conclutions

In conclusion, the intake of fish oil alone or combined with EPO resulted in a higher incorporation of PUFA precursors for the anti-inflammatory lipid mediators in plasma phospholipids. This further induced a significant improvement in the clinical status of patients with rheumatoid arthritis. Further research with a large number of patients over a longer duration is needed to confirm the long-term efficacy of these supplementations.

## Figures and Tables

**Table 1:** Baseline characteristics of the patients with rheumatoid arthritis.

Patients’ Characteristics	Group I	Group II	Group III	*p* Value
	*n* = 20	*n* = 20	*n* = 20	
Age (X ± SD; years)	54 ± 8	57 ± 8	59 ± 7	NS
BMI (kg/m^2^)	26.48 ± 4.15	26.52 ± 4.37	24.65 ± 6.29	NS
Disease duration	6.6 ± 4.0	8.1 ± 2.7	7.2 ± 2.6	NS

Values are expressed as mean ± SD; BMI: body mass index; NS: not statistically significant, assessed by one-way ANOVA.

**Table 2:** Comparisons of clinical and laboratory results in patients at the start and the end of the supplementation.

Patients’ Characteristics	Timing	Group I	Group II	Group III
		*n* = 20	*n* = 20	*n* = 20
CRP (mg/L)	Baseline	12.4 ± 8.2	16.0 ± 18.3	12.7 ± 7.2
	End of study	7.3 ± 2.9 ***	7.1 ± 5.5 ***	6.9 ± 3.5 ***
Tender joint count	Baseline	6.2 ± 2.0	5.4 ± 1.9	5.0 ± 2.0
	End of study	3.3 ± 1.5 ***	4.0 ± 1.4 ***	4.6 ± 1.6
Swollen joint count	Baseline	1.8 ± 1.0	1.5 ± 1.6	1.0 ± 1.3
	End of study	0.8 ± 0.3 ***	0.3 ± 0.8 ***	0.4 ± 0.2 **
VAS (pain)	Baseline	55.7 ± 10.1	59.0 ± 9.1	61.5 ± 8.9
	End of study	46.7 ± 7.1 ***	50.5 ± 7.0 ***	59.3 ± 6.9
ESR, mm/h	Baseline	35.0 ± 24.1	36.7 ± 19.2	33.3 ± 17.1
	End of study	23.2 ± 16.6 ***	19.9 ± 10.8 ***	24.1 ± 13.9 **
DAS 28	Baseline	4.99 ± 0.88	4.76 ± 0.85	4.66 ± 0.80
	End of study	3.91 ± 0.80 ***	3.79 ± 0.72 ***	4.23 ± 0.66

** Significantly different compared to the start value (*p* ≤ 0.01); *** (*p* ≤ 0.001). Values are expressed as mean ± SD; CRP: C-reactive protein; VAS: visual analogue scale; ESR: erythrocyte sedimentation rate; DAS 28: disease activity score 28.

**Table 3:** Fatty acid distribution in plasma phospholipids at baseline and after a 12-week intake of five *Omega*-*3 Cardio* gel capsules daily.

Fatty Acid Distribution	Group I		Statistical Significance
Fatty acid	Baseline	After 12 wk	*p* value
16:0	30.07 ± 5.22	29.47 ± 2.31	NS
16:1 *n*-7	0.57 ± 0.15	0.53 ± 0.23	NS
18:0	18.59 ± 2.82	16.77 ± 2.53	NS
18:1 *n*-9	9.32 ± 1.24	8.05 ± 1.05	NS
18:1 *n*-7	1.99 ± 0.33	1.58 ± 0.25	0.010
18:2 *n*-6LA	23.73 ± 2.43	26.03 ± 2.91	NS
18:3 *n*-3ALA	0.23 ± 0.24	0.21 ± 0.13	NS
18:3 *n*-6GLA	0.00 ± 0.00	0.00 ± 0.00	NS
20:3 *n*-6DGLA	2.84 ± 0.80	2.46 ± 0.94	NS
20:4 *n*-6AA	11.05 ± 3.31	10.22 ± 1.81	NS
20:5 *n*-3EPA	0.32 ± 0.22	1.01 ± 1.02	0.026
22:4 *n*-6	0.45 ± 0.38	0.32 ± 0.13	NS
22:5 *n*-3DPA	0.38 ± 0.17	0.58 ± 0.30	NS
22:6 *n*-3DHA	1.92 ± 0.91	2.74 ± 1.08	0.007
*n*-3	2.85 ± 1.14	4.55 ± 2.26	0.013
*n*-6	38.08 ± 3.80	39.05 ± 3.22	NS
*n*-6/*n*-3	15.47 ± 5.51	10.62 ± 5.07	0.005
SFA	47.17 ± 3.59	46.24 ± 3.97	NS
MUFA	11.89 ± 1.33	10.16 ± 1.34	0.012
PUFA	40.61 ± 3.52	43.13 ± 3.17	0.013

Data are expressed as the mean ± SD; NS: not statistically significant; LA: linoleic acid; GLA: ɤ-linolenic acid; ALA: α-linolenic acid; DGLA: dihomo-ɤ-linolenic acid; AA: arachidonic acid; EPA: eicosapentaenoic acid; DPA: docosapentaenoic acid; DHA: docosahexaenoic acid; SFA: saturated fatty acids; MUFA: monounsaturated fatty acids; PUFA: polyunsaturated fatty acids.

**Table 4:** Fatty acid distribution in plasma phospholipids at baseline and after a 12-week intake of two *Omega*-*3 Cardio* gel capsules and two *Evening Primrose Oil* gel capsules daily.

Fatty Acid Distribution	Group II		Statistical Significance
Fatty acid	Baseline	After 12 week	*p* value
16:0	30.08 ± 5.06	28.85 ± 2.62	NS
16:1 *n*-7	0.56 ± 0.13	0.58 ± 0.26	NS
18:0	16.93 ± 1.83	16.34 ± 1.41	NS
18:1 *n*-9	8.64 ± 1.03	8.44 ± 0.93	NS
18:1 *n*-7	1.75 ± 0.24	1.63 ± 0.23	NS
18:2 *n*-6LA	26.20 ± 1.90	26.30 ± 2.41	NS
18:3 *n*-3ALA	0.22 ± 0.18	0.18 ± 0.15	NS
18:3 *n*-6GLA	0.00 ± 0.00	0.13 ± 0.11	<0.001
20:3 *n*-6DGLA	2.59 ± 0.63	2.67 ± 0.63	NS
20:4 *n*-6AA	10.52 ± 1.81	11.29 ± 2.14	*p* = 0.048
20:5 *n*-3EPA	0.20 ± 0.10	0.49 ± 0.23	*p* < 0.001
22:4 *n*-6	0.37 ± 0.18	0.34 ± 0.13	NS
22:5 *n*-3DPA	0.31 ± 0.08	0.45 ± 0.16	*p* < 0.001
22:6 *n*-3DHA	1.61 ± 0.55	2.22 ± 0.74	*p* = 0.006
*n*-3	2.34 ± 0.65	3.33 ± 1.00	*p* = 0.001
*n*-6	39.69 ± 3.08	40.75 ± 2.86	NS
*n*-6/*n*-3	18.15 ± 5.04	13.50 ± 4.81	*p* = 0.005
SFA	47.01 ± 3.62	45.19 ± 2.80	*p* = 0.039
MUFA	10.56 ± 1.46	10.67 ± 1.12	NS
PUFA	42.04 ± 3.38	44.07 ± 2.96	*p* = 0.020

Data are expressed as the mean ± SD; NS: not statistically significant; Abbreviations as in Table 3.

**Table 5:** Fatty acid distribution in plasma phospholipids at baseline and after 12 weeks without supplementation.

Fatty Acid Distribution	Group III		Statistical Significance
Fatty acid	Baseline	After 12 wk	*p* value
16:0	32.21 ± 3.69	29.67 ± 3.88	NS
16:1 *n*-7	0.72 ± 0.61	0.57 ± 0.12	NS
18:0	16.19 ± 2.40	16.55 ± 1.82	NS
18:1 *n*-9	8.51 ± 1.45	8.35 ± 1.39	NS
18:1 *n*-7	1.69 ± 0.25	1.68 ± 0.30	NS
18:2 *n*-6LA	26.40 ± 3.47	27.49 ± 3.96	NS
18:3 *n*-3ALA	0.21 ± 0.14	0.19 ± 0.23	NS
18:3 *n*-6GLA	0.00 ± 0.00	0.00 ± 0.00	NS
20:3 *n*-6DGLA	2.44 ± 0.78	2.82 ± 0.97	NS
20:4 *n*-6AA	8.98 ± 1.84	9.60 ± 2.90	NS
20:5 *n*-3EPA	0.29 ± 0.18	0.34 ± 0.16	NS
22:4 *n*-6	0.32 ± 0.14	0.29 ± 0.13	NS
22:5 *n*-3DPA	0.32 ± 0.14	0.56 ± 0.69	NS
22:6 *n*-3DHA	1.66 ± 0.69	1.83 ± 0.72	NS
*n*-3	2.46 ± 0.90	2.66 ± 0.81	NS
*n*-6	38.14 ± 3.09	40.03 ± 2.71	NS
*n*-6/*n*-3	17.25 ± 5.51	16.24 ± 3.14	NS
SFA	48.41 ± 3.02	46.26 ± 3.67	NS
MUFA	9.72 ± 2.41	10.61 ± 1.74	NS
PUFA	40.49 ± 4.45	43.60 ± 4.22	NS

Data are expressed as the mean ± SD; Abbreviations as in Table 3.

**Table 6:** Comparisons of fatty acid levels in plasma phospholipids after 12 weeks of intervention.

Fatty Acid	Group I	Group II	Group III
16:0	29.47 ± 2.31	28.85 ± 2.62	29.67 ± 3.88
16:1 *n*-7	0.53 ± 0.23	0.58 ± 0.26	0.57 ± 0.12
18:0	16.77 ± 2.53	16.34 ± 1.41	16.55 ± 1.82
18:1 *n*-9	8.05 ± 1.05	8.44 ± 0.93	8.35 ± 1.39
18:1 *n*-7	1.58 ± 0.25	1.63 ± 0.23	1.68 ± 0.30
18:2 *n*-6LA	26.03 ± 2.91	26.30 ± 2.41	27.49 ± 3.96
18:3 *n*-3ALA	0.21 ± 0.13	0.18 ± 0.15	0.19 ± 0.23
18:3 *n*-6GLA	0.00 ± 0.00	0.13 ± 0.11 ***###	0.00 ± 0.00
20:3 *n*-6DGLA	2.46 ± 0.94	2.69 ± 0.63	2.67 ± 0.77
20:4 *n*-6AA	10.22 ± 1.81	11.29 ± 2.14 *#	9.60 ± 2.90
20:5 *n*-3EPA	1.01 ± 1.02 **	0.49 ± 0.23 *#	0.34 ± 0.16
22:4 *n*-6	0.32 ± 0.13	0.34 ± 0.13	0.29 ± 0.13
22:5 *n*-3DPA	0.58 ± 0.30	0.45 ± 0.16	0.56 ± 0.69
22:6 *n*-3DHA	2.74 ± 1.08 *	2.22 ± 0.74 *	1.83 ± 0.72
*n*-3	4.55 ± 2.26 **	3.33 ± 1.00 *	2.66 ± 0.81
*n*-6	39.05 ± 3.22	40.75 ± 2.86	40.03 ± 2.71
*n*-6/*n*-3	10.62 ± 5.07 **	13.50 ± 4.81 *	16.24 ± 3.14
SFA	46.24 ± 3.97	45.19 ± 2.80	46.26 ± 3.67
MUFA	10.16 ± 1.34	10.67 ± 1.12	10.61 ± 1.74
PUFA	43.13 ± 3.17	44.07 ± 2.96	43.60 ± 4.22

* Significantly different from the group III (*p* ≤ 0.05); ** (*p* ≤ 0.01); *** (*p* ≤ 0.001). # Significantly different from the group I # (*p* ≤ 0.05); ### (*p* ≤ 0.001). Assessed by one-way ANOVA followed by the Tukey post hoc test. Data are expressed as the mean ± SD; Abbreviations as in Table 3.

## References

[1] Széles L., Töröcsik D., Nagy L. (2007). PPAR gamma in immunity and inflammation: Cell types and diseases. Biochim. Biophys. Acta.

[2] Giera M., Ioan-Facsinay A., Toes R., Gao F., Dalli J., Deelder A.M., Serhan C.N., Mayboroda O.A. (2012). Lipid and lipid mediator profiling of human synovial fluid in rheumatoid arthritis patients by means of LC-MS/MS. Biochim. Biophys. Acta.

[3] Woo S.J., Lim K., Park S.Y., Jung M.Y., Lim H.S., Jeon M.G., Lee S.I., Park B.H. (2015). Endogenous conversion of *n*-6 to *n*-3 polyunsaturated fatty acids attenuates K/BxN serum-transfer arthritis in fat-1 mice. J. Nutr. Biochem..

[4] Bannenberg G., Arita M., Serhan C.N. (2007). Endogenous receptor agonists: Resolving inflammation. Sci. World J..

[5] Calder P.C. (2006). *n*-3 polyunsaturated fatty acids, inflammation and inflammatory diseases. Am. J. Clin. Nutr..

[6] Barden A.E., Moghaddami M., Mas E., Phillips M., Cleland L.G., Mori T.A. (2016). Specialised pro-resolving mediators of inflammation in inflammatory arthritis. Prostaglandins Leukot. Essent. Fatty Acids.

[7] Volker D., Fitzgerald P., Major G., Garg M. (2000). Efficacy of fish oil concentrate in the treatment of rheumatoid arthritis. J. Rheumatol..

[8] Calder P.C. (2002). Dietary modification of inflammation with lipids. Proc. Nutr. Soc..

[9] Mori T.A., Beilin L.J. (2004). Omega-3 fatty acids and inflammation. Curr. Atheroscler. Rep..

[10] Barden A.E., Mas E., Mori T.A. (2016). *n*-3 Fatty acid supplementation and proresolving mediators of inflammation. Curr. Opin. Lipidol..

[11] Dalli J., Colas R.A., Serhan C.N. (2013). Novel *n*-3 immunoresolvents: Structures and actions. Sci. Rep..

[12] Chiang N., Serhan C.N. (2006). Cell-cell interaction in the transcellular biosynthesis of novel omega-3-derived lipid mediators. Methods Mol. Biol..

[13] Serhan C.N., Petasis N.A. (2011). Resolvins and protectins in inflammation resolution. Chem. Rev..

[14] Hong S., Lu Y. (2013). Omega-3 fatty acid-derived resolvins and protectins in inflammation resolution and leukocyte functions: Targeting novel lipid mediator pathways in mitigation of acute kidney injury. Front. Immunol..

[15] Pullman-Mooar S., Laposata M., Lem D., Holman R.T., Leventhal L.J., DeMarco D., Zurier R.B. (1990). Alteration of the cellular fatty acid profile and the production of eicosanoids in human monocytes by gamma-linolenic acid. Arthritis Rheum..

[16] Brzeski M., Madhok R., Capell H.A. (1991). Evening primrose oil in patients with rheumatoid arthritis and side-effects of non-steroidal anti-inflammatory drugs. Br. J. Rheumatol..

[17] Leventhal L.J., Boyce E.G., Zurier R.B. (1993). Treatment of rheumatoid arthritis with gammalinolenic acid. Ann. Intern. Med..

[18] Leventhal L.J., Boyce E.G., Zurier R.B. (1994). Treatment of rheumatoid arthritis with blackcurrant seed oil. Br. J. Rheumatol..

[19] Zurier R.B., Rossetti R.G., Jacobson E.W. (1996). Gamma-Linolenic acid treatment of rheumatoid arthritis. A randomized, placebo-controlled trial. Arthritis Rheum..

[20] Cameron M., Gagnier J.J., Little C.V., Parsons T.J., Blümle A., Chrubasik S. (2009). Evidence of effectiveness of herbal medicinal products in the treatment of arthritis. Part 2: Rheumatoid arthritis. Phytother. Res..

[21] Aursnes M., Tungen J.E., Vik A., Colas R., Cheng C.Y., Dalli J., Serhan C.N., Hansen T.V. (2014). Total synthesis of the lipid mediator PD1n-3 DPA: Configurational assignments and anti-inflammatory and pro-resolving actions. J. Nat. Prod..

[22] Spite M., Clària J., Serhan C.N. (2014). Resolvins, specialized proresolving lipid mediators, and their potential roles in metabolic diseases. Cell Metab..

[23] Serhan C.N., Dalli J., Colas R.A., Winkler J.W., Chiang N. (2015). Protectins and maresins: New pro-resolving families of mediators in acute inflammation and resolution bioactive metabolome. Biochim. Biophys. Acta.

[24] Liu C., Hume A.L., Krinsky D.L. (2015). Fish oil supplements for rheumatoid arthritis: Some beneficial effects. Pharm. Today.

[25] Aletaha D., Neogi T., Silman A.J., Funovits J., Felson D.T., Bingham C.O., Birnbaum N.S., Burmester G.R., Bykerk V.P., Cohen M.D. (2010). 2010 Rheumatoid arthritis classification criteria: An American College of Rheumatology/European League against Rheumatism collaborative initiative. Arthritis Rheum..

[26] DAS 28 calculator V1.1-beta by Alfons and Michiel. http://www.umcn.nl.DAS28.

[27] Petrović S., Takić M., Arsić A., Vučić V., Drakulić D., Milošević M., Glibetić M. (2014). Effect of Sex Hormones on Plasma Phospholipid Fatty Acid Composition in Intact Rats and Rats with Bilaterally Occluded Carotid Arteries. Physiol. Res..

[28] Christopherson S.W., Glass R.L. (1969). Preparation of milk methyl esters by alcoholysis in an essentially nonalcoholic solution. J. Daily Sci..

[29] Arab L., Akbar J. (2002). Biomarkers and the measurement of fatty acids. Public Health Nutr..

[30] Cvetković Z., Vučić V., Cvetković B., Petrović M., Ristić-Medić D., Tepšić J., Glibetić M. (2010). Abnormal fatty acid distribution of the serum phospholipids of patients with non-Hodgkin lymphoma. Ann. Hematol..

[31] Ristic-Medic D., Takic M., Vucic V., Kostic N., Glibetic M. (2013). Abnormalities in serum phospholipids fatty acid profile in patients with alcoholic liver currhosis- a pilot study. J. Clin. Biochem. Nutr..

[32] Arsić A., Vučić V., Tepšić J., Mazić S., Djelić M., Glibetić M. (2012). Altered plasma and erythrocyte phospholipid fatty acid profile in elite female water polo and football players. Appl. Physiol. Nutr. Metab..

[33] Tepsic J., Vucic V., Arsic A., Mazic S., Djelic M., Glibetic M. (2013). Unfavourable plasma and erythrocyte phospholipid fatty acid profile in elite amateur boxers. Eur. J. Sport Sci..

[34] Vučić V. (2013). The role of dietary polyunsaturated fatty acids in inflammation. Serbian J. Exp. Clin. Res..

[35] Murphy R.A., Mourtzakis M., Chu Q.S., Baracos V.E., Reiman T., Mazurak V.C. (2011). Supplementation with fish oil increases first-line chemotherapy efficacy in patients with advanced nonsmall cell lung cancer. Cancer.

[36] Wang C., Harris W.S., Chung M., Lichtenstein A.H., Balk E.M., Kupelnick B., Jordan H.S., Lau J. (2006). *n*-3 Fatty acids from fish or fishoil supplements, but not alpha-linolenic acid, benefit cardiovascular disease outcomes in primary- and secondary- prevention studies: A systematic review. Am. J. Clin. Nutr..

[37] Berbert A.A., Kondo C.R., Almendra C.L., Matsuo T., Dichi I. (2005). Supplementation of fish oil and olive oil in patients with rheumatoid arthritis. Nutrition.

[38] Das Gupta A.B., Hossain A.K., Islam M.H., Dey S.R., Khan A.L. (2009). Role of omega-3 fatty acid supplementation with indomethacin in suppression of disease activity in rheumatoid arthritis. Bangladesh Med. Res. Counc. Bull..

[39] Dawczynski C., Schubert R., Hein G., Müller A., Eidner T., Vogelsang H., Basu S., Jahreis G. (2009). Long-term moderate intervention with *n*-3 long-chain PUFA supplemented dairy products: Effects on pathophysiological biomarkers in patients with rheumatoid arthritis. Br. J. Nutr..

[40] Calder P.C. (2015). Marine omega-3 fatty acids and inflammatory processes: Effects, mechanisms and clinical relevance. Biochim. Biophys. Acta.

[41] Lee Y.H., Bae S.C., Song G.G. (2012). Omega-3 polyunsaturated fatty acids and the treatment of rheumatoid arthritis: A meta-analysis. Arch. Med. Res..

[42] Barham J.B., Edens M.B., Fonteh A.N., Johnson M.M., Easter L., Chilton F.H. (2000). Addition of eicosapentaenoic acid to gamma-linolenic acid supplemented diets prevents serum arachidonic acid accumulation in humans. J. Nutr..

[43] Bhangle S., Kolasinski S.L. (2011). Fish oil in rheumatic diseases. Rheum. Dis. Clin. N. Am..

[44] Miles E.A., Calder P.C. (2012). Influence of marine *n*-3 polyunsaturated fatty acids on immune function and a systematic review of their effects on clinical outcomes in rheumatoid arthritis. Br. J. Nutr..

[45] Rajaei E., Mowla K., Ghorbani A., Bahadoram S., Bahadoram M., Dargahi-Malamir M. (2015). The Effect of Omega-3 Fatty Acids in Patients With Active Rheumatoid Arthritis Receiving DMARDs Therapy: Double-Blind Randomized Controlled Trial. Glob. J. Health Sci..

[46] Olendzki B.C., Leung K., Van Buskirk S., Reed G., Zurier R.B. (2011). Treatment of rheumatoid arthritiswith marine and botanical oils: Influence on serum lipids. Evid. Based Complement. Altern. Med..

[47] Klein K., Gay S. (2015). Epigenetics in rheumatoid arthritis. Curr. Opin. Rheumatol..

[48] Fenton J.I., Hord N.G., Ghosh S., Gurzell E.A. (2013). Immunomodulation by dietary long chain omega-3 fatty acids and the potential for adverse health outcomes. Prostaglandins Leukot. Essent. Fatty Acids.

[49] Sandoo A., Veldhuijzen van Zanten J.J., Metsios G.S., Carroll D., Kitas G.D. (2011). Vascular function and morphology in rheumatoid arthritis: A systematic review. Rheumatology (Oxf.).

[50] Harris W.S., Von Schacky C. (2004). The Omega-3 Index: A new risk factor for death from coronary heart disease?. Prev. Med..

[51] Jabbar R., Saldeen T. (2006). A new predictor of risk for sudden cardiac death. Ups. J. Med. Sci..

[52] Dawczynski C., Hackermeier U., Viehweger M., Stange R., Springer M., Jahreis G. (2011). Incorporation of *n*-3 PUFA and γ-linolenic acid in blood lipids and red blood cell lipids together with their influence on disease activity in patients with chronic inflammatory arthritis—A randomized controlled human intervention trial. Lipids Health Dis..

[53] Aviña-Zubieta J.A., Choi H.K., Sadatsafavi M., Etminan M., Esdaile J.M., Lacaille D. (2008). Risk of cardiovascular mortality in patients with rheumatoid arthritis: A meta-analysis of observational studies. Arthritis Rheum..

[54] Meune C., Touzé E., Trinquart L., Allanore Y. (2009). Trends in cardiovascular mortality in patients with rheumatoid arthritis over 50 years: A systematic review and meta-analysis of cohort studies. Rheumatology (Oxf.).

